# Cnm of *Streptococcus mutans* is important for cell surface structure and membrane permeability

**DOI:** 10.3389/fcimb.2022.994014

**Published:** 2022-09-13

**Authors:** Shuhei Naka, Daiki Matsuoka, Kana Goto, Taro Misaki, Yasuyuki Nagasawa, Seigo Ito, Ryota Nomura, Kazuhiko Nakano, Michiyo Matsumoto-Nakano

**Affiliations:** ^1^ Department of Pediatric Dentistry, Okayama University Graduate School of Medicine, Dentistry and Pharmaceutical Sciences, Okayama, Japan; ^2^ Division of Nephrology, Seirei Hamamatsu General Hospital, Hamamatsu, Japan; ^3^ Department of Nursing, Faculty of Nursing, Seirei Christopher University, Hamamatsu, Japan; ^4^ Department of General Internal Medicine, Hyogo College of Medicine, Nishinomiya, Japan; ^5^ Department of Internal Medicine, Japan Self-Defense Iruma Hospital, Iruma, Japan; ^6^ Department of Pediatric Dentistry, Division of Oral infection and Disease Control, Osaka University Graduate School of Dentistry, Suita, Japan

**Keywords:** *Streptococcus mutan*s, collagen-binding protein, membrane permeability, cell structure, RNA-seq

## Abstract

*Streptococcus mutans*, a Gram-positive facultative anaerobic bacterium, is a major pathogen of dental caries. The protein Cnm of *S. mutans* is involved in collagen binding, but its other biological functions are unknown. In this study, a Cnm-deficient isogenic mutant and a complementation strain were generated from a Cnm-positive *S. mutans* strain to help determine the properties of Cnm. Initially, comparison of the cell surface structure was performed by electron microscopy, which demonstrated that Cnm appears to be localized on the cell surface and associated with a protruding cell surface structure. Deep RNA sequencing of the strains revealed that the defect in Cnm caused upregulated expression of many genes related to ABC transporters and cell-surface proteins, while a few genes were downregulated. The amount of biofilm formed by the Cnm-defective strain increased compared with the parental and complemented strains, but the biofilm structure was thinner because of elevated expression of genes encoding glucan synthesis enzymes, leading to increased production of extracellular polysaccharides. Particular antibiotics, including bacitracin and chloramphenicol, had a lower minimum inhibitory concentration for the Cnm-defective strain than particular antibiotics, including bacitracin and chloramphenicol, compared with the parental and complemented strains. Our results suggest that *S. mutans* Cnm is located on the cell surface, gives rise to the observed protruding cell surface, and is associated with several biological properties related to membrane permeability.

## Introduction

*Streptococcus mutans*, a Gram-positive facultative anaerobic bacterium, is a major pathogen related to dental caries (Bowen, 2016). The major surface proteins of *S. mutans* involved in the pathogenicity of dental caries include the 190-kDa protein antigen (PA), glucosyltransferases (GTFB/C/D), and glucan binding proteins (GbpA/B/C/D), all of which can be detected with high frequency in clinical isolates of *S. mutans* (Matsumoto-Nakano, 2018). Biofilm formation by *S. mutans* is established *via* two distinct adhesion mechanisms, the sucrose-dependent and sucrose-independent mechanisms. GTFs and Gbps are major pathogenic proteins in the sucrose-dependent mechanisms, while PA functions in the sucrose-independent mechanism. The importance of the GTFs in cariogenicity, particularly the products of the *gtfB* and *gtfC* genes, has been established by many researchers (Bowen and Koo, 2011). Simultaneous synthesis of glucans by GTFB and GTFC is essential for establishment of a matrix that enhances the coherence of bacterial cells and adherence to tooth surfaces, allowing for formation of high-density biofilms (Tamesada et al., 2004; Xiao and Koo, 2010). However, GTFD, which synthesizes water-soluble glucans rich in α-1, 6-glucosidic linkages, has been detected in culture supernatant (Hanada and Kuramitsu, 1989). Binding of *S. mutans* to glucans formed *in situ* is mediated by the presence of GTF enzymes and Gbps (Banas and Vickerman, 2003). GbpC appears to be anchored to the cell wall, is somewhat similar to members of the Spa family of oral streptococcal proteins, and is involved in rapid dextran-dependent aggregation in stressful growth conditions (Sato et al., 2004). These proteins participate in bacterial adherence to tooth surface and coordinate the biofilm formation.

Cnm, a cell-wall anchored protein related to binding to type I collagen, is also identified in approximately 10% of clinical isolates of *S. mutans* (Sato et al., 2004). Cnm has been found to function in the sucrose-independent adhesion mechanism in the biofilm formation involved in colonization of oral tissues, and in adhesion in extraoral infections. Cnm is an important virulence factor in the onset or aggravation of systemic disease (Nakano et al., 2011; Nomura et al., 2013; Naka et al., 2014; Naka et al., 2016; Naka et al., 2018).

Aside from these proteins, there are many membrane transporters in the plasma membrane of *S. mutans*, which are involved in the absorption of substances for the growth of the bacteria (Ajdic et al., 2002), and export and import of various molecules (Ajdic et al., 2007). Upregulation of and/or the presence of proteins associated with several types of ABC transporter are associated with the efflux of antibiotics in several multidrug-resistant species (Nikaido, 2009). Alteration of the cell-wall architecture affects resistance to antimicrobial compounds (Nikaido, 2009; Fischer et al., 2011). Therefore, the lack of cell surface proteins and/or cell-wall associated proteins may lead to multiple alterations of pathogenic factors of *S. mutans*, in addition to impaired adherence and biofilm formation.

The purpose of this study was to analyze the localization and function of Cnm, to identify its contribution to the virulence of *S. mutans*.

## Materials and methods

### *S. mutans* strains and culture conditions

*S. mutans* strain SN74 (serotype *e*, Cnm-positive) was isolated from the oral cavity of a patient with a severe IgA nephropathy (Naka et al., 2020). The strain was cultured on Mitis-Salivarius (MS; Becton Dickinson, Franklin Lakes, NJ, USA) agar plates containing bacitracin (0.2 U/ml; Sigma Chemical Co., St. Louis, MO, USA) or in brain heart infusion (BHI; Becton Dickinson) broth or Todd Hewitt (TH; Becton Dickinson) broth.

### Preparation and purification of Recombinant Cnm protein

The DNA fragment encoding the entire Cnm protein in *S. mutans* TW871, a strain isolated from blood of a patient with infective endocarditis-complicated subarachnoid hemorrhage, were amplified using specific primer set *cnm* (pGEX6p-1) (**Table 1**) and PrimeSTAR^®^ MAX DNA Polymerase (TaKaRa Bio, Shiga, Japan), and ligated into vector pGEX6p-1 (Cytiva, Tokyo, Japan). The PCR amplification reaction was performed in a thermal cycler (iCycler; Bio-Rad, Hercules, CA, USA) with the following cycling parameters: initial denaturation at 95°C for 4 min; 30 cycles of 95°C for 30 s, 60°C for 30 s, and 72°C for 2 min; and a final extension at 72°C for 7 min. The DNA fragments were assembled using the GeneArt^®^ Seamless Cloning and Assembly Kit (Thermo Fisher Scientific, Waltham, MA). rCnm protein was obtained by transforming the plasmid into *Escherichia coli* BL21 (DE3) (Nippon Gene, Tokyo, Japan), as described previously (Takashima et al., 2015). The cells were grown in Luria-Bertani broth to the mid-exponential phase at 37°C, then recombinant protein expression was induced with 1.0 mM isopropyl-1-thio-β-D-galactopyranoside (Wako Pure Chemical Corporation, Osaka, Japan) at 16°C for 24 h. Cells were harvested by centrifugation and pelleted cells were resuspended in phosphate-buffered saline (PBS). Lysozyme (0.25 mg/ml; Roche Diagnostics, Basel, Switzerland) was added and the mixture was incubated at 4°C for 30 min, followed by intermittent sonication on ice for 3 min with a 1 min rest period; and this process was repeated three times. The supernatant was obtained by centrifugation, and applied to a glutathione-Sepharose 4B column (particle size: 90 µm; Cytiva) and eluted with 10 mM glutathione buffer (50 mM Tris-HCl, 10 mM glutathione, pH8.0) at 4°C. Finally, the purified glutathione *S*-transferase (GST)-fusion protein was freeze‐dried and used in this study.

**Table 1:** PCR primers used in this study.

Name	Sequence (5'→3')	Reference
cnm-(pGEX6P1)-F	CTG TTC CAG GGG CCC ATG AAA AGA AAA GGT TTA CGA AGA C	This study
cnm-(pGEX6P1)-R	GCA GAT CGT CAG TCA TCA GCT ATG ATA TTT ACG GTA AAC T	
pGEX6P1-cnm-F	AAA TAT CAT AGC TGA TGA CTG ACG ATC TGC CTC GCG CGT T	This study
pGEX6P1-cnm-R	ACC TTT TCT TTT CAT GGG CCC CTG GAA CAG AAC TTC CAG A	
pGEX6P1C-F	ATG GAC CCA ATG TGC CTG GAT GCG T	This study
pGEX6P1C-R	CCG GGA GCT GCA TGT GTC AGA GGT T	
cnm-1F	GAC AAA GAA ATG AAA GAT GT	Nakano et al., 2004
cnm-1R	GCA AAG ACT CTT GTC CCT GC	
cnm-erm-F	GAA GGA ATG CTT GCA TGA AC	Nakano et al., 2004
cnm-erm-R	GAC TCA TAG CAT TCT TTC CTC CCG	
cnm-*Hin*dIII-F	GCG AAG CTT ATG AAA AGA AAA GGT TTA CGA	Naka et al., 2016
cnm-*Bam*HI-R	CGG GGA TCC TAT CAG TTA TGA TGT TTA CGG	
cnm-CF	CTA CCG TTT TCT ACT ATA AGA CTG GGG	Naka et al., 2016
cnm-CR	CCT TCT TGA CCG CGA TAA GAC TCA CTG CCA	

### Generation of antiserum against rCnm

Cnm antiserum was generated by intramuscular administration of rCnm in rabbits, as described previously (Matsumoto-Nakano et al., 2007; Nomura et al., 2012). Briefly, antiserum against Cnm was generated by injecting rabbits (New Zealand White; Oriental Yeast Co. Ltd., Tokyo, Japan) with four intramuscular injections of purified rCnm (1200 μg) emulsified with a block copolymer adjuvant (Titer-Max Gold; CytRx Co., Atlanta, GA, USA) every 2 weeks for a total of 8 weeks. The antibody titer of each antiserum sample was confirmed by an enzyme-linked immunosorbent assay (ELISA) using rCnm.

### Construction of Cnm-defective isogenic mutant and complemented strains

A Cnm-defective mutant strain of *S. mutans* was generated according to the method of Nakano et al., 2004. Initially, the full length of the *cnm* gene was amplified by PCR using the template of DNA from *S. mutans* TW871, AmpliTaq^®^ (Life Technologies, Grand Island, NY, USA) and primers cnm-1F and cnm-1R (**Table 1**) designed based on the *cnm* sequence of *S. mutans* TW295 (GenBank accession number AB469913). The amplified DNA fragments were ligated into pGEM-T Easy Vector (Promega Co., Madison, WI, USA) to produce plasmid pTN11 carrying the *cnm* gene. Next, the erythromycin (EM) resistance gene derived from pVA838 (Macrina et al., 1983) was amplified by PCR using primers cnm-ermF and cnm-ermR (**Table 1**) and inserted into pTN11 in the middle of the *cnm* gene (which had been cut with *Bsm*I) to produce pTN12. We then transformed pTN12 into *S. mutans* strain SN74 using the protocol of Tobian and Macrina (1982) and produced Cnm-defective mutant strain SN74CND in which the *cnm* gene was inactivated.

To generate a complemented strain, first, the open reading frame region of the *cnm* gene (GenBank accession number: AB469914) of *S. mutans* strain TW871 was amplified by PCR using the template of DNA from *S. mutans* TW871 and primers cnm-*Hind*III-F and cnm-*Bam*HI-R (**Table 1**). The resulting DNA was digested with restriction enzymes *Hind*III and *Bam*HI and ligated into pDL278 (Dunny et al., 1991) to produce a plasmid containing the *cnm* gene. Then, this plasmid was transduced into *S. mutans* strain SN74CND to produce complemented strain SN74CNDcomp.

To verify the mutant strains, chromosomal DNA was extracted and PCR was performed using primers cnm-1F and cnm-1R to confirm the full-length sequence of the *cnm* gene (containing the EM resistance gene where applicable). Reverse transcription PCR (RT-PCR) (Nomura et al., 2005) was used to confirm *cnm* gene expression. The collagen binding activity of the strains was measured by the method described below.

### Bacterial growth rates

Overnight cultures of *S. mutans* were inoculated into TH medium with the cultures performed in duplicate. Growth curves were determined by monitoring OD_550_ values at 1-h intervals using a spectrophotometer (GE Healthcare, Fairfield, CT, USA).

### Collagen-binding properties of strains

Collagen-binding properties were analyzed according to the protocol described by Waterhouse and Russell (2006) with some modifications. Type I collagen (0.002–2 mg collagen in 0.25 M acetic acid; Sigma) was coated onto 96-well tissue culture plates (Beckton Dickinson) and incubated overnight at 4°C, then the plates were washed and blocked with bovine serum albumin (BSA) solution. Next, the wells were washed and reacted with overnight cultures of *S. mutans* [1 × 10^8^–1 × 10^10^ colony-forming units (CFU)] for 3 h at 37°C, then adherent cells were washed and fixed with 25% formaldehyde at room temperature for 30 min. After washing, the adherent cells were stained with 0.05% crystal violet (Wako) for 1 min and washed, then the dye was dissolved by adding 7% acetic acid (200 µl) before determining OD_595_ values. The results for each strain were expressed as a percentage compared with the binding of parental strain SN74, which was defined as 100%.

### Scanning electron microscopy

SEM was performed according to the protocol described by Yuan et al. (2021) to observe morphologic changes of tested strains. Overnight cultures of *S. mutans* were harvested and pre-fixed with 2% glutaraldehyde and 2% paraformaldehyde at 4°C for 16 h then washed with 0.1 M phosphate buffer (pH 7.4), fixed with 2% osmium tetroxide for 1.5 h, and washed with 0.1 M phosphate buffer (pH 7.4). Then, samples were dehydrated using an ethanol gradient, immersed in t-butyl alcohol for 30 min, and dried with CO_2_ for 2 h. The prepared specimens were placed on aluminum stubs, coated with osmium (HPC-IS, Vacuum Device, Ibaraki, Japan) and observed by SEM (S-4500, Hitachi, Tokyo, Japan).

### Transmission electron microscopy

TEM was performed according to the method of Naka et al. (2020). For pre-fixation, cell specimens were immersed in 0.1 M PBS, pH 7.4, containing 2% glutaraldehyde and 2% paraformaldehyde for 16–18 h. Post-fixation was performed in 2% osmium tetroxide for 1.5 h. After washing with PBS, the specimens were dehydrated in a graded ethanol series and embedded in low viscosity resin (Spurr resin, Polysciences). Then, 80-nm sections were prepared using an ultramicrotome (EM-UC7; Leica, Tokyo, Japan) and stained with uranyl acetate and lead citrate. Specimens were observed by TEM (H-7650, Hitachi, Tokyo, Japan).

### Immunogold TEM

Sections (80-nm) were mounted on a 100-mesh nickel grid, and incubated with PBS containing 10% goat serum (GEMINI Bio, San Carlos, CA) and 1% BSA. Sections were incubated with anti-Cnm antibody overnight at 4°C and washed with PBS containing 0.1% BSA >5 times. Then, they were incubated with gold colloid [Anti-IgG (H+L), Rabbit, Goat-Poly, Gold 15 nm; BBI solutions, Crumlin, UK] conjugate with goat anti-rat IgG antibody (Biolegend, SanDiego, CA, USA) and washed three times with PBS containing 0.1% BSA and then washed once with distilled water. Specimens were finally fixed with 2% glutaraldehyde.

### RNA extraction and deep sequencing

Total RNAs of *S. mutans* strains SN74, SN74CND, and SN74CNDcomp were purified as described previously (Matsumoto-Nakano and Kuramitsu, 2006). The quality of enriched mRNA samples was determined using an Agilent Bioanalyzer (Agilent Technologies, Santa Clara, CA, USA).

cDNA libraries were constructed from the enriched mRNA samples by using an NEBNext Ultra Directional RNA Library Prep Kit for Illumina and NEBNext Multiplex Oligonucleotides for Illumina (New England BioLabs, Ipswich, MA, USA), following the protocol of the supplier. Deep sequencing was performed by the NextGen DNA Sequencing Core Laboratory of ICBR at the University of Florida (Gainesville, FL, USA). Read mapping was performed on a Galaxy server hosted by the research computing center at the University of Florida, using Map with Bowtie for Illumina (version 1.1.2).

### Gene expression analysis

To confirm the validity of results obtained using RNA-Seq, conventional real-time quantitative reverse transcription PCR (qRT-PCR) was employed to measure changes in the mRNA levels of open reading frames. SuperScript III^®^ Reverse Transcriptase (Invitrogen, Carlsbad, CA, USA) and random primers (Promega) were used to obtain cDNA from DNA-free RNA from *S. mutans* strains SN74 and SN74CND. PCR was then performed on DNA (as a positive control), cDNA, and MilliQ water (as a negative control), with primers for either the 16S ribosomal RNA (rRNA) gene or a specific gene (**Table 2**). The qRT-PCR reaction was conducted using SYBR^®^ Green (Bio-Rad Laboratories, Hercules, CA, USA) in an iCycler thermal cycler (Bio-Rad), according to the manufacturer’s protocol. The mRNA expression values were quantified by the ΔΔCT method using 16s rRNA as the internal control.

**Table 2:** Primers used for qRT-PCR in this study.

Name	Sequence (5'→3')	Reference
16SrRNA-F	CAG CGC AGC TGA TAG CTG TTT GT CT	Matsumoto-Nakano and Kuramitsu, 2006
16SrRNA-R	CTG CTG GCA AAT TCG CTT ACT TG	
*gtfB*-F	GAT GGG TGA CAG TAT CTG TTGC	Inagaki et al., 2013
*gtfB-*R	GAG CTA CTG ATT GTC GTT ACTG	
*gtfC*-F	GAT GCT TCT GGG TTC CAA GCT	Inagaki et al., 2013
*gtfC*-R	CGA TTA CGA ACT TCA TTT CCGG	
*gtfD*-F	GTT TGA TTA CCT TGG GCA CCA CAA CAT TGG	Inagaki et al., 2013
*gtfD*-R	ACG TTT GCC TGA CTT TGG GTC TGC GTT TGT	
*gbpA*-F	GTG ACT AGT CTA GCT CTG GCT GCG ATA TTG	This study
*gbpA*-R	CAG CGT TAG CAC TGT TAT TTT CTA CAG ATG	
*gbpB*-F	TCA GCA GTT TTA GTG AGT GGT GTA ACT CTT	This study
*gbpB*-R	AAT TTG TTG ACC CAA AGT AGC AGA CTG AGC	
*gbpC*-F	CCT ACT GCT GAT ACA CAA GCA TCA GAA CCG	This study
*gbpC*-R	GAG CTT CAG TTT CAG TGA CTT CTA AAC CTT	

### Fluorescence efflux measurement

Fluorescence measurements were performed using the method described by Ocaktan et al. (1997) with some modifications. Strains SN74, SN74CND, and SN74CNDcomp were grown to OD_550_ = 0.4 in TH medium and pelleted by centrifugation at 2400 × *g* for 10 min at 4°C. The cells were then washed with 10 mM NaCl and 50 mM sodium phosphate (pH 7.0), and resuspended in the same buffer. Before fluorescence probe labeling, cultures were adjusted to an optical density of 0.2 at 600 nm.

One milliliter of adjusted sample was labeled with *N*-phenyl-2-naphtylamine (NPN) reacted at a final concentration of 5 or 10 µg/ml and incubated with light shielding for 30 min. NPN is a fluorescence polarization probe that is a useful indicator of phase transitions in the dispersion of intact membranes (Trauble and Overath, 1973). Following incubation, labeled cultures were centrifuged at 2400 × *g* for 10 min at 4°C, and the resultant pellets were washed twice with 500 µl of 10 mM NaCl and 50 mM sodium phosphate (pH 7.0). Thereafter, 100-µl samples were plated in 96-well plates (Nunc, Roskilde, Denmark), and fluorescence was determined using a Twinkle LB970 fluorometer (Berthold Technologies GmbH & Co. KG, Bad Wildbad, Germany) with excitation at 355 nm and emission at 460 nm.

### Determination of minimum inhibitory concentrations

MICs of antibiotics were determined using a microbroth dilution method previously described by the Clinical and Laboratory Standards Institute (CLSI) (de Jong et al., 2020). Briefly, tested strains in 3 ml of Muller-Hinton broth (Becton Dickinson) supplemented with 4.25 µM MgCl_2_ and 90 µM CaCl_2_ and containing two fold serial dilution of antibiotics were placed in sterile 13 mm × 100 mm test tubes. Test strains were cultured in BHI broth at 37°C for 18 h and washed, then 5×10^5^ CFU were added to the tubes containing the antimicrobial agents and incubated at 37°C for 18 h. Breakpoints were set based on CLSI guidelines (de Jong et al., 2020).

### Quantitative and structural analyses of biofilms

The ability of strains to form biofilms was evaluated by growing cells in wells of 96-well polystyrene microtiter plates according to previously described protocol (Ardin et al., 2014). TH medium (diluted 1:4) containing 0.1% sucrose was mixed with a pre-grown cell suspension (OD_550_ = 2.0), and then 100 µl of the samples was inoculated into the individual wells. The plates were incubated anaerobically at 37°C for 48 h. After incubation, formed biofilms were stained with 1% crystal violet (Sigma) for 15 min at room temperature. The plate was next rinsed six times with sterile distilled water to remove loosely bound bacteria, dried, then fixed with 95% ethanol. Stained biofilms were quantified by measuring the absorbance at 570 nm using an ELISA microplate reader (Thermo Fisher Scientific, Waltham, MA, USA). Three independent experiments were performed in triplicate.

Quantitative and structural analyses of biofilms were performed using confocal laser scanning microscopy (CLSM), according to the protocol described by Kuboniwa et al. (2006). The tested strains were separately cultured in 10 ml of TH medium overnight at 37°C, then centrifuged at 2400 × *g* for 5 min at 4°C and the cells were washed with distilled water. Next, the cells were labeled with hexidium iodide (Invitrogen) and incubated in the dark for 15 min at room temperature. Each cell suspension was adjusted to OD_600_ = 0.1 in chemically-defined medium (CDM) supplemented with 0.5% sucrose (van de Rijn and Kessler, 1980), and then 100 µl of each suspension was added to a Lab-Tek Chambered #1.0 Borosilicate Coverglass System with eight chambers (Nunc) that had been coated with filtered 25% human saliva to allow biofilm formation. The chambers were incubated at 37°C with light shielding in an anaerobic chamber for 24 h, after which the CDM supplemented with 0.5% sucrose was removed and 100 µl of PBS was added.

Imaging was performed using an LSM 510 confocal laser scanning microscope (version 4.2, Carl Zeiss MicroImaging Co., Ltd., Jena, Germany) at a laser wavelength of 543 nm. Biofilm images of each sample were acquired from three random positions. The obtained confocal microscopy images were analyzed using ImageJ software for Macintosh (version 10.2, National Institute of Health, Bethesda, MD, USA, USA).

### Statistical Analysis

Statistical analyses were performed using GraphPad Prism 8 software (Graph Pad Software Inc., San Diego, CA). All data are presented as the mean ± standard deviation of the means. Differences in fluorescence intensity and amount of biofilm formation were assessed using analysis of variance with Bonferroni’s correction. Expression levels of genes (*gtfB*, *gtfC*, *gtfD*, *gbpA*, *gbpB*, and *gbpC*) were compared using analysis of variance with the Mann-Whitney U test. *P*-values <0.05 were considered statistically significant.

## Results

### Construction of Cnm-deficient mutant strain

**Figure 1:**
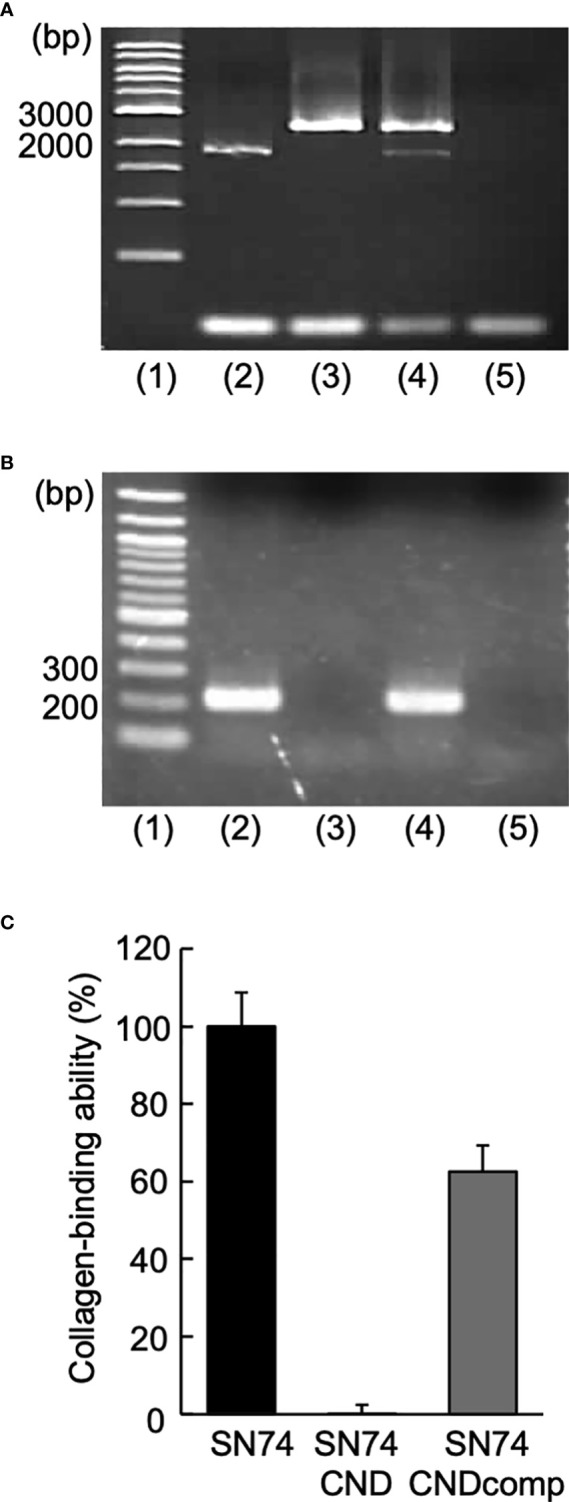
Construction of *Streptococcus mutans* strains SN74CND and SN74CNDcomp. **(A)** Confirmation of chromosomal DNA [lane 1: 1-kbp DNA ladder, 2: strain SN74 (the parental strain), 3: strain SN74CND (the *cnm* deletion strain), 4: strain SN74CNDcomp (complementation of the *cnm* deletion strain), and 5: MilliQ water as a negative control). **(B)** Confirmation of cDNA using reverse transcription-PCR (lane 1: 100-bp DNA ladder; 2: strain SN74; 3: strain SN74CND; 4: strain SN74CNDcomp; 5: ultrapure water as a negative control). **(C)** Collagen binding ability. The results for each strain are expressed as a percentage relative to the binding ability of parental strain SN74, which was defined as 100%. Data are presented as the mean ± SD from five independent experiments.

Primer extension analysis was used to determine the *cnm* transcription sites in *S. mutans* strain SN74 using primers cnm-1F and cnm-1R (**Table 1**). Agarose gel electrophoresis of the PCR products showed an amplified band of approximately 1728 bp (**Figure 1A**). RT-PCR did not amplify the *cnm* gene from cDNA obtained from Cnm-defective mutant strain SN74CND. In contrast, the *cnm* gene was amplified from cDNA obtained from strain SN74CNDcomp and the band was the same size as that from the parental strain SN74 (**Figure 1B**). The growth rates of strains SN74, SN74CND, and SN74CNDcomp were determined. There was no difference in the growth of strain SN74 and SN74CNDcomp whereas that of strain SN74CND was slightly reduced **(Supplementary Figure 1**). In addition, typical rough colonies were observed on MS-agar plates for strains SN74, SN74CND, and SN74CNDcomp (**Supplementary Figure 2**). Strain SN74CND could scarcely bind collagen, whereas strain SN74CNDcomp bound collagen at approximately 60% of the level of the parental strain SN74 (**Figure 1C**).

### Generation and purification of rCnm and Cnm antiserum

**Figure 2:**
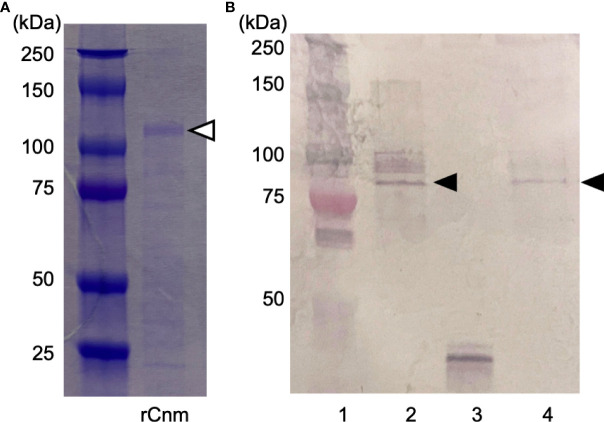
Generation and purification of recombinant Cnm and Cnm antiserum. **(A)** SDS-PAGE (Coomassie blue staining). The Cnm-glutathione S-transferase fusion protein is indicated with a white arrowhead. **(B)** Western blot analysis of expression of Cnm (lane 1: protein molecular weight markers; 2: strain SN74; 3: strain SN74CND; 4: strain SN74CNDcomp). Cnm is indicated with a black arrowhead.

Purified GST-rCnm fusion protein was subjected to sodium dodecyl sulfate-polyacrylamide gel electrophoresis stained with Coomassie blue (**Figure 2A**). The expected value of the molecular weight of the GST-rCnm fusion proteins was approximately 120 kDa (**Figure 2A**).

To confirm the immunoreactive specificity of our rCnm antiserum, we used cells of strains SN74, SN74CND, and SN74CNDcomp. Western blot analysis produced a positive band in whole cell extracts of strains SN74 and SN74CNDcomp, whereas no such band was observed when using extracts of cells of strain SN74CND (**Figure 2B**).

### Bacterial cell surface conditions and Cnm

**Figure 3:**
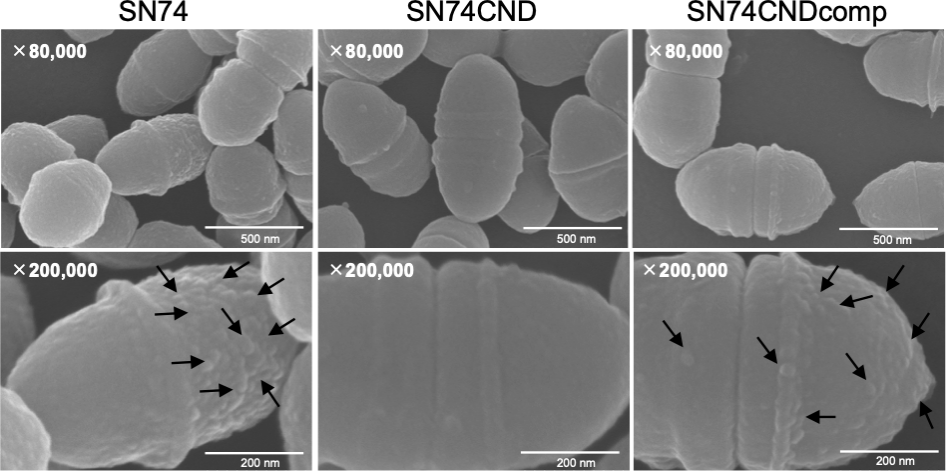
Scanning electron microscopic images of *S. mutans* strains SN74, SN74CND, and SN74CNDcomp. Scale bar of the upper image, 500 nm; scale bar of the lower image, 200 nm; magnification of the upper image, ×80,000; magnification of the lower image, ×200,000. The arrows indicate the bumpy structures.

**Figure 4:**
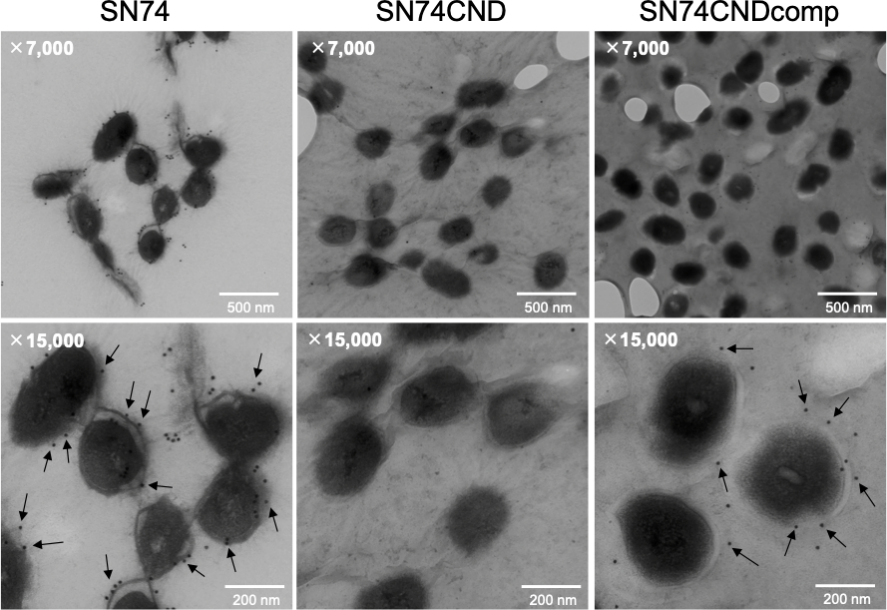
Immunogold transmission electron microscopic images of *S. mutans* strains SN74, SN74CND, and SN74CNDcomp. Scale bar of the upper image, 500 nm; scale bar of the lower image, 200 nm; magnification of the upper image, ×7,000; magnification of the lower image, ×15,000. The arrows indicate gold colloidal particles.

SEM images of *S. mutans* cells were very different for strains SN74 and SN74CND (**Figure 3**). In SEM images of strain SN74, a bumpy structure of the cell surface was observed, while nothing was observed on the cell surface of strain SN74CND (i.e., it was smooth). The bumpy structure of the cell surface was recovered in strain SN74CNDcomp. Therefore, we observed SEM images of other clinical isolates of *S. mutans*, and found that strains TW871, TW295, and OMZ175, which have Cnm, had bumpy structures on the cell surface similar to those of strain SN74 (**Supplementary Figure 3A**). However, bumpy structures were not observed on the cell surfaces of strains MT8148, UA159, and GS5, each of which lacks Cnm (**Supplementary Figure 3B**). To confirm that the protruding structures involved Cnm, we performed immunogold TEM imaging using the anti-Cnm antibody (**Figure 4**). In the TEM images of strain SN74, numerous adherent gold colloidal particles were observed around the bacterial cell wall, but these were not observed in images of strain SN74CND (**Figure 4**). TEM images of strain SN74CNDcomp showed recovery of gold colloidal particle attachment around the bacterial cell wall (**Figure 4**). In addition, TEM images also showed differences in the peptidoglycan layer between strains SN74 and SN74CND (**Figure 4**). The peptidoglycan layer of strain SN74 appeared clear and smooth. In contrast, the peptidoglycan layer of strain SN74CND was obscure. These results indicated that the protrusion from the cell surface involved Cnm and the presence of Cnm affects the bacterial surface conditions.

### RNA-seq to identify genes regulated by Cnm

**Figure 5:**
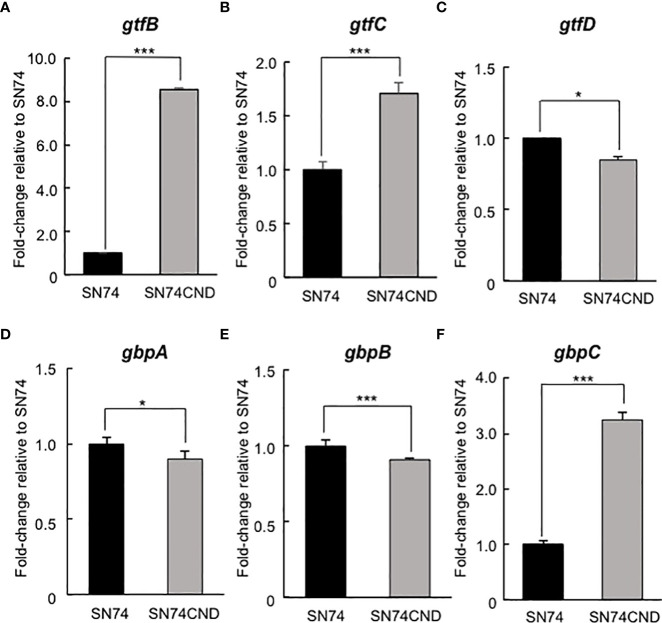
Comparison of gene expression levels in strains SN74 and SN74CND by real-time quantitative reverase transcription PCR. **(A)** *gtfB*, **(B)** *gtfC*, **(C)** *gtfD*, **(D)** *gbpA*, **(E)** *gbpB*, **(F)** *gbpC*. The mRNA expression values were quantified by the ΔΔCT method using 16s rRNA as the internal control. Data are presented as the mean ± SD from five independent experiments. Statistical significance was determined using analysis of variance with the Mann-Whitney U test. *P < 0.05, ***P < 0.001.

The cut-off for designating a gene as being differentially expressed was a change in mRNA level of at least twofold (up- or downregulated). Gene expression in strain SN74CND compared with that in strain SN74 is shown in **Tables 3**–**5**. Expression of transcripts in strain SN74CND was significantly altered compared with the parental strain SN74. Interestingly, the lack of Cnm altered the expression levels of genes related to cell surface proteins, including GTFs and Gbps, which are involved in biofilm formation. In contrast, the absence of Cnm did not influence expression of PA, which is also a cell-anchored protein. Focusing on genes showing the highest levels of differential expression that were related to biofilm formation, a subset of these results was confirmed using qRT-PCR (**Figure 5**). *S. mutans* produces three types of GTF, encoded by *gtfB*, *gtfC*, and *gtfD*, respectively, and four types of Gbps encoded by *gbpA*, *gbpB*, *gbpC*, and *gbpD*, respectively, which are components associated with the adhesion phase of caries development (Matsumoto-Nakano, 2018). The expression of *gtfB*, *gtfC*, and *gbpC* in strain SN74CND was significantly higher than that in strain SN74 (*P* < 0.001). In contrast, the expression levels of *gtfD*, *gbpA*, and *gbpB* in strain SN74CND were significantly lower than those in strain SN74 (*P* < 0.05, *P* < 0.05, and *P* < 0.001, respectively). The expression level of *gbpD* did not change. Furthermore, the absence of Cnm affected the expression of genes related to transporters, competence, and stress tolerance. Therefore, Cnm has a strong relationship with mature biofilm formation.

Table 3List of genes whose expression was upregulated (>2-fold) in strain SN74CND compared with parental strain SN74 by RNA-Sequencing.

**Table:** 

Cell surface protein-related genes				
*Gene name*	*Description*	*NCBI Gene ID*	*Locus Tag*	*Fold-change*
*gtfB*	Glucosyltransferase-I	1028336	SMU_1004	17.031
*gbpC*	Glucan-binding protein GbpC	1028662	SMU_1396	6.575
*gtfC*	Glucosyltransferase-SI	1028343	SMU_1005	3.395

**Table:** 

ABC transporter-related genes				
*Gene name*	*Description*	*NCBI Gene ID*	*Locus Tag*	*Fold-change*
*SMU_934*	Amino acid ABC transporter permease	1029516	SMU_934	49.079
*SMU_936*	Amino acid ABC transporter ATP-binding protein	1028285	SMU_936	46.026
*SMU_935*	Amino acid ABC transporter permease	1028288	SMU_935	45.114
*SMU_933*	Amino acid ABC transporter substrate-binding protein	1028287	SMU_933	37.424
*SMU_651c*	ABC transporter substrate-binding protein	1028079	SMU_651c	29.796
*SMU_652c*	Nitrate ABC transporter ATP-binding protein	1029588	SMU_652c	20.165
*SMU_653c*	ABC transporter permease	1028071	SMU_653c	19.048
*ptnC*	PTS system mannose-specific transporter subunit IIC	1029084	SMU_1878	10.726
*SMU_1551c*	ABC transporter ATP-binding protein	1028790	SMU_1551c	9.088
*SMU_1093*	ABC transporter permease	1029552	SMU_1093	4.797
*SMU_1879*	PTS system mannose-specific transporter subunit IID	1029083	SMU_1879	4.778
*SMU_1571*	MsmK-like ABC transporter ATP-binding protein	1028806	SMU_1571	4.614
*SMU_1094*	ABC transporter ATP-binding protein	1028415	SMU_1094	3.998
*malG*	Maltose ABC transporter permease	1028808	SMU_1570	3.837
*mtlA1*	PTS system mannitol-specific transporter subunit IIBC	1028492	SMU_1185	3.792
*SMU_241c*	Amino acid ABC transporter ATP-binding protein	1027838	SMU_241c	3.676
*SMU_1899*	ABC transporter ATP-binding protein/permease (fragment)	1029097	SMU_1899	3.498
*SMU_1067c*	ABC transporter permease	1029512	SMU_1067c	3.304
*SMU_803c*	ABC transporter ATP-binding protein	1029385	SMU_803c	3.297
*SMU_311*	PTS system sorbitol (glucitol) transporter subunit IIC2	1028201	SMU_311	3.273
*Gene name*	*Description*	*NCBI Gene ID*	*Locus Tag*	*Fold-change*
*SMU_1897*	ABC transporter ATP-binding protein	1029101	SMU_1897	3.138
*SMU_312*	PTS system sorbitol phosphotransferase transporter subunit IIBC	1027880	SMU_312	3.057
*SMU_313*	PTS system sorbitol-specific transporter subunit IIA	1029420	SMU_313	2.925
*SMU_872*	PTS system fructose-specific transporter subunit IIABC	1028238	SMU_872	2.920
*copA*	Copper-transporting ATPase	1028623	SMU_426	2.886
*nrgA*	Ammonium transporter	1028890	SMU_1658	2.865
*SMU_1006*	ABC transporter ATP-binding protein	1028342	SMU_1006	2.842
*SMU_998*	ABC transporter periplasmic ferrichrome-binding protein	1029602	SMU_998	2.834
*SMU_1119c*	Sugar ABC transporter permease	1028435	SMU_1119c	2.814
*SMU_1905c*	Bacteriocin secretion protein	1029103	SMU_1905c	2.748
*scrA*	PTS system sucrose-specific transporter subunit IIABC	1029057	SMU_1841	2.701
*SMU_1118c*	ABC sugar transporter, permease	1028430	SMU_1118c	2.609
*opuBc*	Choline ABC transporter, osmoprotectant binding protein	1028413	SMU_1095	2.592
*trk*	Potassium uptake protein TrkA	1028798	SMU_1562	2.544
*pacL*	Cation-transporting P-type ATPase PacL	1028803	SMU_1563	2.540
*mtlA2*	PTS system mannitol-specific transporter subunit IIA	1028483	SMU_1183	2.524
*SMU_675*	PTS system transporter protein EI	1028092	SMU_675	2.503
*ptnA*	PTS system mannose-specific transporter subunit IIAB	1029081	SMU_1877	2.447
*opuBa*	Choline transporter ABC transporter ATP-binding protein	1028412	SMU_1096	2.421
*SMU_1163c*	ABC transporter ATP-binding protein	1028468	SMU_1163c	2.394
*sloC*	ABC transporter metal binding lipoprotein	1029755	SMU_184	2.381
*SMU_1041*	ABC transporter ATP-binding protein	1028375	SMU_1041	2.381
*SMU_1068c*	ABC transporter ATP-binding protein	1029549	SMU_1068c	2.328
*pdhC*	Branched-chain alpha-keto acid dehydrogenase E2 subunit	1028680	SMU_1421	2.319
*lacE*	PTS system lactose-specific transporter subunit IIBC	1028733	SMU_1491	2.306
*oadB*	Oxaloacetate decarboxylase, sodium ion pump subunit	1028350	SMU_1017	2.285
*SMU_1934c*	Cobalt ABC transporter ATP-binding protein	1029130	SMU_1934c	2.216
*SMU_1898*	ABC transporter ATP-binding protein/permease	1029098	SMU_1898	2.199
*sgaT*	PTS system ascorbate-specific transporter subunit IIC	1029474	SMU_270	2.088

**Table:** 

Signal transmission-related genes				
*Gene name*	*Description*	*NCBI Gene ID*	*Locus Tag*	*Fold-change*
*cysK*	Cysteine synthetase A	1027990	SMU_496	11.035
*pyrH*	Uridylate kinase	1028858	SMU_1625	6.254
*celR*	Transcriptional regulator	1028830	SMU_1599	5.497
*brpA*	Transcriptional regulator	1029569	SMU_410	4.619
*SMU_1657c*	Nitrogen regulatory protein PII	1028889	SMU_1657c	3.904
*copZ*	Copper chaperone	1027946	SMU_427	2.545
*SMU_1548c*	Histidine kinase	1028787	SMU_1548c	2.398

Table 4List of genes whose expression was downregulated (>2-fold) in strain SN74CND compared with parental strain SN74 by RNA-Sequencing.

**Table:** 

Cell surface protein-related genes				
*Gene name*	*Description*	*NCBI gene ID*	*Locus tag*	*Fold-change*
*gtfD*	Glucosyltransferase-S	1028270	SMU_910	−2.405
*gbpB*	Secreted antigen GbpB/SagA	1029610	SMU_22	−2.131
**Cell wall protein-related genes**				
*Gene name*	*Description*	*NCBI gene ID*	*Locus tag*	*Fold-change*
*mreC*	Cell shape-determining protein MreC	1029606	SMU_20	−4.180
*pbp2a*	Membrane carboxypeptidase, penicillin-binding protein 2a	1029145	SMU_1949	−3.529
*wapA*	Cell wall-associated protein WapA	1028331	SMU_987	−2.671
*mreD*	Cell shape-determining protein MreD	1029607	SMU_21	−2.508
*pbp2x*	Penicillin-binding protein 2X	1028367	SMU_455	−2.438
*pbp2b*	Penicillin-binding protein 2b	1028046	SMU_597	−2.073

**Table:** 

ABC transporter-related genes				
*Gene name*	*Description*	*NCBI gene ID*	*Locus tag*	*Fold-change*
*comF*	Late competence protein	1027989	SMU_498	−15.538
*comYB*	ABC transporter ComYB	1029176	SMU_1985	−12.007
*ptcB*	PTS system cellobiose transporter subunit IIB	1028835	SMU_1600	−11.644
*comYA*	ABC transporter ATP-binding protein ComYA; late Competence gene	1029164	SMU_1987	−9.926
*SMU_2149c*	Cobalt transporter ATP-binding subunit	1029326	SMU_2149c	−3.115
*malX*	Maltose ABC transporter substrate-binding protein	1028671	SMU_1568	−2.836
*SMU_100*	PTS system sorbose transporter subunit IIB	1029681	SMU_100	−2.743
*SMU_1178c*	Amino acid ABC transporter ATP-binding protein	1028485	SMU_1178c	−2.646
*SMU_2150c*	Cobalt transporter ATP-binding subunit	1029324	SMU_2150c	−2.609
*SMU_1938c*	ABC transporter permease	1029134	SMU_1938c	−2.597
*adcC*	ABC transporter ATP-binding protein	1029185	SMU_1994	−2.582
*SMU_683*	ATP-binding protein	1029361	SMU_683	−2.459
*SMU_815*	Amino acid ABC transporter substrate-binding protein	1029428	SMU_815	−2.436
*SMU_1179c*	Amino acid ABC transporter permease	1029561	SMU_1179c	−2.425
*SMU_731*	ABC transporter ATP-binding protein	1028135	SMU_731	−2.372
*bglP*	PTS system beta-glucoside-specific transporter subunit II	1028324	SMU_980	−2.359
*potA*	Spermidine/putrescine ABC transporter ATP-binding protein	1028319	SMU_973	−2.272
*glnQ*	Amino acid ABC transporter ATP-binding protein	1028758	SMU_1519	−2.233
*SMU_567*	Glutamine ABC transporter permease	1029364	SMU_567	−2.125
*SMU_1078c*	ABC transporter ATP-binding protein	1028403	SMU_1078c	−2.039
*adcB*	ABC transporter zinc permease	1029187	SMU_1993	−2.014
*SMU_2035*	Bacteriocin immunity protein	1029223	SMU_2035	−2.007

**Table:** 

Signal transmission-related genes				
*Gene name*	*Description*	*NCBI gene ID*	*Locus tag*	*Fold-change*
*SMU_626*	Competence protein	1028064	SMU_626	−10.558
*comEA*	Competence protein	1029564	SMU_625	−7.482
*comYC*	Competence protein ComYC	1029177	SMU_1984	−5.753
*ropA*	Trigger factor	1029670	SMU_91	−5.553
*ssb2*	Single-stranded DNA-binding protein	1029162	SMU_1967	−3.747
*SMU_228*	Alkaline-shock protein-like protein	1027831	SMU_228	−3.661
*SMU_499*	Late competence protein	1027992	SMU_499	−3.627
*Gene name*	*Description*	*NCBI gene ID*	*Locus tag*	*Fold-change*
*dnaB*	Chromosome replication protein	1029121	SMU_1922	−3.609
*SMU_44*	DNA mismatch repair protein	1029632	SMU_44	−3.387
*SMU_644*	Competence protein/transcription factor	1029517	SMU_644	−3.068
*SMU_1923c*	Transcriptional regulator NrdR	1029118	SMU_1923c	−3.050
*SMU_2060*	LysR family transcriptional regulator	1029235	SMU_2060	−2.860
*dnaC*	Replicative DNA helicase	1029303	SMU_2138	−2.800
*comYD*	Competence protein ComYD	1029175	SMU_1983	−2.787
*ruvA*	Holliday junction DNA helicase RuvA	1029268	SMU_2088	−2.744
*SMU_1034c*	Site-specific tyrosine recombinase XerS	1028083	SMU_1034c	−2.711
*recU*	Holliday junction-specific endonuclease	1027970	SMU_469	−2.486
*SMU_2078c*	Holliday junction resolvase-like protein	1029249	SMU_2078c	−2.356
*cinA*	Competence damage-inducible protein A	1029264	SMU_2086	−2.311
*SMU_1780*	Recombination regulator RecX	1028996	SMU_1780	−2.270

**Table 5:** List of genes showing no significant change in expression in strain SN74CND compared with parental strain SN74 by RNA-Sequencing.

Cell surface protein-related genes				
*Gene name*	*Description*	*NCBI gene ID*	*Locus tag*	*Fold-change*
*gtfA*	Sucrose phosphorylase GtfA	1028245	SMU_881	1.438
*gbpD*	Glucan-binding protein D	1028158	SMU_772	1.322
*SMU_1039c*	Lipopolysaccharide glycosyltransferase	1028363	SMU_1039c	1.250
*SMU_1434c*	Glycosyltransferase	1028717	SMU_1434c	1.070
*gbpA*	Glucan-binding protein GbpA	1029286	SMU_2112	1.029
*SMU_833*	Glycosyltransferase	1029490	SMU_833	1.000
*rgpE*	Glycosyltransferase	1029496	SMU_829	−1.959
Cell wall protein-related genes				
*Gene name*	*Description*	*NCBI gene ID*	*Locus tag*	*Fold-change*
*ftsH*	Cell division protein FtsH	1029422	SMU_15	1.677
*ftsY*	cell division protein FtsY	1029340	SMU_744	1.636
*wapE*	cell wall protein WapE	1029536	SMU_1091	1.331
*dexB*	Dextran glucosidase DexB	1028248	SMU_883	1.294
*ftsA*	Cell division protein FtsA	1029599	SMU_551	−1.052
*SMU_1449*	Fibronectin/fibrinogen-binding protein	1028699	SMU_1449	−1.064
*pbpX*	Penicillin-binding protein, class C; fmt-like protein	1028247	SMU_889	−1.082
*ftsW*	Cell division protein FtsW	1028117	SMU_713	−1.131
*murM*	Peptidoglycan branched peptide synthesis protein MurM	1028122	SMU_717	−1.182
*pbp1a*	Penicillin-binding protein 1a; membrane carboxypeptidase	1027967	SMU_467	−1.217
*murN*	Peptidoglycan branched peptide synthesis protein MurN	1027827	SMU_716	−1.262
*spaP*	Cell surface antigen SpaP	1028055	SMU_610	−1.419
*SMU_1279c*	Cell shape determining protein	1028567	SMU_1279c	−1.454
*dltD*	Extramembranal protein DltD	1028924	SMU_1688	−1.621
*divIVA*	Cell division protein DivIVA	1029404	SMU_557	−1.730

### Analysis of exocytosis

**Figure 6:**
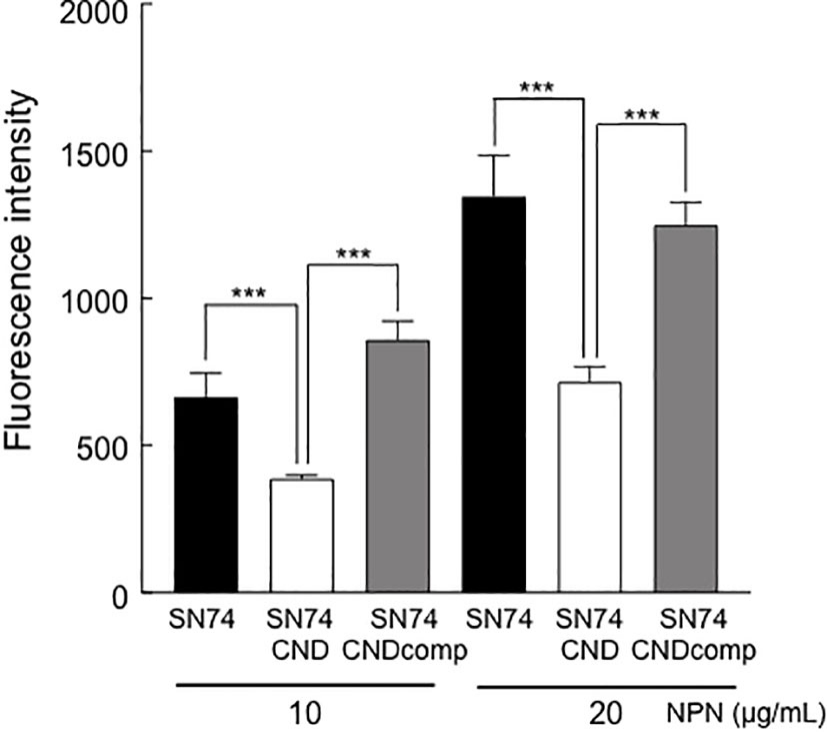
Analysis of exocytosis in strains SN74, SN74CND, and SN74CNDcomp. Fluorescence of the cells was examined in the presence of different concentrations of the fluorescent probe *N*-phenyl-2-naphtylamine. Data are presented as the mean ± SD from five independent experiments. Statistical significance was determined using analysis of variance with Bonferroni’s correction. ***P < 0.001.

NPN is an uncharged lipophilic probe that can be used to monitor the fluidity of the lipid layer (Nieva-Gomez and Gennis, 1977). Addition of 10 or 20 μg/ml NPN led to an increase in fluorescence intensity of strains SN74, SN74CND, and SN74CNDcomp (**Figure 6**). However, the intensity of strain SN74CND was significantly less than that of strain SN74 (*P* < 0.001), suggesting a decrease to less than half of the amount of exported molecules. The wild-type phenotype was fully restored in strain SN74CNDcomp (*P* < 0.001). Our analysis of exocytosis with NPN showed fewer molecules being released from structures in the plasma membrane in strain SN74CND than in strains SN74 and SN74CNDcomp. These results indicate that the lack of Cnm may affect transmembrane processes involving molecular export and import.

### Evaluation of MICs

MICs were evaluated nine antibiotics with strains SN74, SN74CND, and SN74CNDcomp. The MICs of for doripenem and ofloxacin for strain SN74CND were higher, and those of bacitracin and chloramphenicol were lower, than for the other strains (**Table 6**). Furthermore, strain SN74 was sensitive to clindamycin, but strain SN74CND and SN74CNDcomp were resistant to CLDM. CLSI proposed in 2004 that the erythromycin gene product methylates the ribosome leading to resistance to CLDM. An erythromycin-resistance gene was inserted in the nucleotide sequence of the strain SN74CND produced in this study, suggesting that resistance to CLDM may be acquired through resistance to erythromycin.

**Table 6:** Antibiotic resistance test; Minimum Inhibitory Concentration.

	SN74	SN74CND	SN74CNDcomp
PCG	0.008	0.008	0.008
CFPM	<0.004	<0.004	<0.004
DRPM	0.03	0.06	0.03
VCM	1	1	1
BAC	128	64	128
TC	0.5	0.5	0.5
OFLX	1	2	1
CP	4	2	4
CLDM	<0.004	>32	>32

The MICs of these strains were determined using a macro-diffusion broth method previously described by Clinical and Laboratory Standards Institute (CLSI) (2012).

PCG; Penicillin G, CFPM; Cefpime; DRPM; Doripenem, VCM; Vancomycin, TC; Tetracycline, OFLX; Ofloxacin, CP; Chloramphenicol, CLDM; Clindamycin.

### Biofilm formation

**Figure 7:**
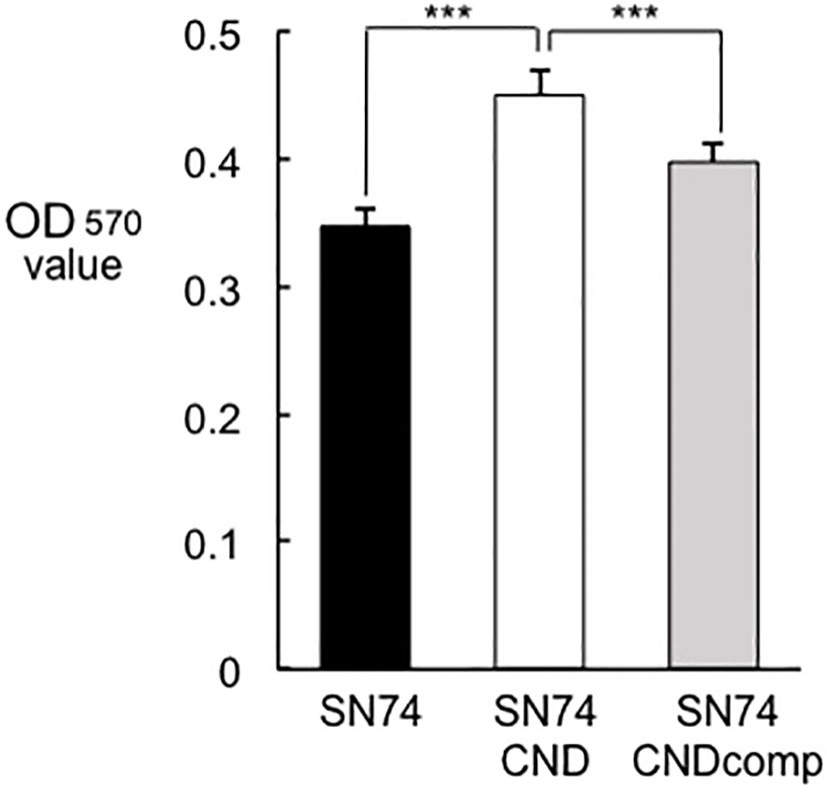
Amount of biofilm formation by strains SN74, SN74CND, and SN74CNDcomp. Biofilm formation by *S. mutans* strains SN74, SN74CND, and SN74CNDcomp cultured in 1.0% sucrose-added Todd Hewitt broth. The quantity of biofilm formation was determined from the OD570 value following crystal violet staining. Data are presented as the mean ± SD from five independent experiments. Statistical significance was determined using analysis of variance with Bonferroni’s correction. ***P < 0.001.

**Figure 8:**
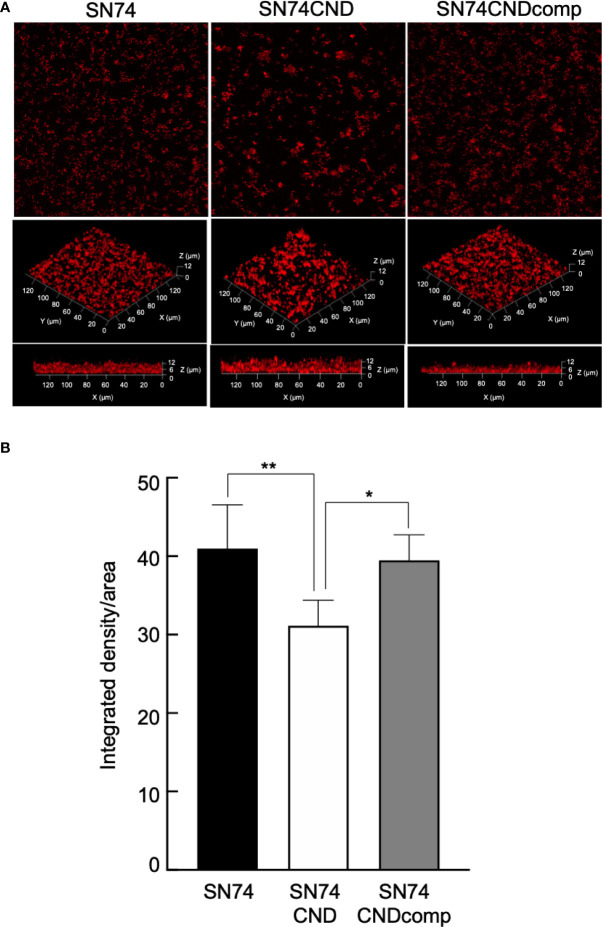
Observation of biofilm formation using confocal laser scanning microscopy **(A)**. Confocal laser scanning microscopic images of *S. mutans* strains SN74, SN74CND and SN74CNDcomp. **(B)** Densitometric analysis of biofilms generated by strains SN74, SN74CND, and SN74CNDcomp. Biofilm images for each sample were acquired using three random positions and three independent experiments were performed in triplicate for each strain. Data are presented as the mean ± SD. Statistical significance was determined using analysis of variance with Bonferroni’s correction. *P < 0.05; **P < 0.01.

Cnm-deficient mutant strain SN74CND formed significantly more biofilm than strain SN74 (*P* < 0.001) (**Figure 7**). The Cnm-complemented strain SN74CNDcomp formed significantly less biofilm than strain SN74CND (*P* < 0.001), and a comparable amount to the parental strain SN74 (**Figure 7**). The biofilms formed by strain SN74CND had greater thickness than those formed by SN74. However, biofilms formed by strain SN74CND showed both small and large amorphous microcolonies (**Figure 8A**). The morphology of the biofilms of strain SN74CNDcomp was the same as that of the parental strain SN74 (**Figure 8A**). Biofilm density and thickness were quantified using ImageJ software, and the biofilms of strain SN74CND showed significantly lower values than those for the parental strain SN74 (*P* < 0.01). The values for strain SN74CNDcomp were significantly higher than those for strain SN74CND (*P* < 0.05) and comparable to those for the parental strain SN74 (**Figure 8B**).

## Discussion

Although *S. mutans* is a major causative pathogen of dental caries, it is not associated with binding to soft tissues. Recently, however, Cnm has been characterized as a novel LPXTG (leucine, proline, X, threonine, and glycine, where X is any amino acid) motif-anchored protein of *S. mutans*, which contributes to adhesion to and invasion of soft tissues, such as arterial and venous endothelial cells (Nomura et al., 2013). However, the biological properties of Cnm are yet to be fully elucidated. In this study, we investigated the role of Cnm in pathogenicity of *S. mutans*.

SEM images and immunogold TEM images indicated that Cnm is a cell surface protein and that the bumpy structures on the surfaces of cells possessing Cnm are possibly Cnm. Surface components for bacterial contact with environmental niches are involved in bacterial adhesion to and invasion of a host, and escape from host defense systems (Bucior et al., 2012; Chagnot et al., 2013). In Gram-negative bacteria, these virulence factors are pilus or fimbrial structures, but they are seldom observed in micrographs of Gram-positive bacteria (Proft and Baker, 2009; Bucior et al., 2012). This surface structure, Cnm, is an important virulence factor of *S. mutans*.

The ability of bacteria to colonize and persist within their respective environments is strongly influenced by the cell surface composition and resultant functional properties (Crowley and Brady, 2016). Protein translocation and folding pathways are pivotal for the proper insertion of integral membrane proteins, and for the secretion, maturation, and function of fully externalized molecules (Yuan et al., 2010; Forster and Marquis, 2012; Schneewind and Missiakas, 2014). *S. mutans* lacking the extracellular chaperone/foldase PrsA showed auto-aggregation and altered cell surface properties (Guo et al., 2013). In a mutant strain deficient in RopA, a ribosome-associated chaperone, the expression of GTFB and GTFD was lower compared with that in the parental strain (Wen et al., 2005). Furthermore, inactivation of the *gbpA* gene altered the expression of *gtfB* and *gtfC* (Hazlett et al., 1998). Therefore, the lack of cell surface proteins may cause a change of cell structure. In the present study, the deletion of Cnm resulted in changes in the expression levels of many genes, in particular, many membrane transporter-associated genes (**Tables 3**–**5**). Membrane transporters are common and comprise one of the largest protein families (Linton, 2007). *S. mutans* is subject to a number of environmental fluctuations, such as nutrient availability, aerobic-to-anaerobic transitions, and pH changes (Lemos and Burne, 2008). The transport systems are one of the methods used for surviving these fluctuations, and *S. mutans* contains >280 genes associated with various transport systems involved in the uptake of ions, molecules, and carbohydrates (Ajdic et al., 2002). The upregulation of membrane transporter genes could promote these functions.

In the present study, the expression of many transporter-related genes was changed by deletion of Cnm. We also examined exocytosis of our strains using a fluorescent polarization probe, NPN, which can used to monitor outer membrane permeabilization (Helander, 2000). The amounts of exocytosis by strain SN74CND decreased significantly when compared with strains SN74 and SN74CNDcomp (**Figure 6**). In addition, the MICs of chloramphenicol and bacitracin toward strain SN74CND were lower than those toward strains SN74 and SN74CNDcomp. These findings suggest that Cnm blocks uptake and diffusion of some molecules, which may be one strategy used by *S. mutans* to survive changes in its environment.

In addition, Cnm may be a negative regulator of *gtf* and *gbp* genes because the expression levels of *gtfB*, *gtfC*, and *gbpC* increased in strain SN74CND relative to those in the parental strain. In the present study, the quantity of biofilm formation by strain SN74CND increased when compared with the amounts formed by strains SN74 and SN74CNDcomp (**Figure 7**). However, the structure of the biofilm formed by strain SN74CND showed both small and large amorphous microcolonies and it was not stable and firm compared with the biofilms formed by strains SN74 and SN74CNDcomp (i. e., it was tinner, and less dense) (**Figure 8**). Upregulation of *gtfB*, *gtfC*, and *gbpC* in strain SN74CND would exacerbate glucan synthesis and cell aggregation by GTFB, GTFC, and GbpC. As a result, many microcolonies formed and the density of the density of the resulting biofilm was decreased. Our results suggest that the lack of Cnm influences biofilm formation by *S. mutans*. In other words, Cnm is related to biofilm formation (extracellular polymeric substance synthesis and binding) in *S. mutans* by regulating several genes such as those encoding GTFs and Gbps.

Although Cnm is not associated with the development of dental caries, Cnm has been reported to enter the bloodstream and to be associated with various systemic diseases. Recently, exacerbation mechanisms of systemic diseases such as cerebral hemorrhage, ulcerative colitis and nonalcoholic steatohepatitis caused by *S. mutans* harboring Cnm have been clarified (Nakano et al., 2011; Kojima et al., 2012; Naka et al., 2014; Naka et al., 2016; Naka et al., 2018). *S. mutans* possessing Cnm is able to induce infective endocarditis when entering the bloodstream during invasive dental procedures such as tooth extraction (Naka et al., 2016). Cnm protein is involved in adhesion to and invasion of vascular endothelial cells, and is considered to be an important factor in the pathogenesis of their systemic disease (Abranches et al., 2011; Nomura et al., 2013). In the present study, Cnm was found to be located at the cell surface of *S. mutans* and showed a protrusive structure. These results suggest that Cnm may function as an antigen or like fimbrillin of *Porphyromonas gingivalis*.

In summary, Cnm located on the cell surface of *S. mutans*, appears to show a protruding structure, and is associated with several biological properties related to membrane permeability and the regulation of surface and transporter protein genes in the bacterium.

## Data availability statement

The original contributions presented in the study are included in the article/**Supplementary Material**. Further inquiries can be directed to the corresponding author.

## Author contributions

SN and DM designed the study under the supervision of MM-N and KN. YN, TM, and SI advised on MIC analysis. Statistical analyses were performed by SN, TM, and SI. Data interpretation was performed by SN, TM, KG, DM, and RN. SN, KN, and MM-N wrote the manuscript and all authors approved the final version. All authors contributed to the article and approved the submitted version.

## Acknowledgments

This work was supported by funding from JSPS KAKENHI (grant numbers JP17K09721, JP18H03010, JP19K10098, JP20K10225, and JP21K08242). We thank Masumi Furutani, Urata Haruo and Moemi Tsukano (Central Research Laboratory, Okayama University Graduate School of Medicine, Dentistry and Pharmaceutical Sciences) for assistance with electron microscopic analyses. We thank Edanz Group (https://en-author-services.edanzgroup.com/ac) for editing a draft of this manuscript.

## Conflict of interest

The authors declare that the research was conducted in the absence of any commercial or financial relationships that could be construed as a potential conflict of interest.

## Publisher’s note

All claims expressed in this article are solely those of the authors and do not necessarily represent those of their affiliated organizations, or those of the publisher, the editors and the reviewers. Any product that may be evaluated in this article, or claim that may be made by its manufacturer, is not guaranteed or endorsed by the publisher.

## Supplementary material

Supplementary Figure 1 Bacterial growth rates of strains SN74, SN74CND, and SN74CNDcomp. ○ SN74, Δ SN74CND, SN74CNDcomp. There were significant differences in the values between strains SN74 and SN74CND (****P* < 0.001), and between strains SN74CNDcomp and SN74CND (^###^ *P* < 0.001). Data are presented as the mean ± SD from five independent experiments. Statistical significance was determined using analysis of variance with Bonferroni’s correction.

Supplementary Figure 2 Colony morphology of *S. mutans* strains SN74, SN74CND, and SN74CNDcomp.

Supplementary Figure 3 Scanning electron microscopic images of *S. mutans* strains. A. Cnm-positive *S. mutans* strains TW871, TW295, and OMZ175. Scale bar of the upper image, 500 nM; scale bar of the lower image, 200 nM; magnification of the upper image, ×80,000; magnification of the lower image, ×200,000. B. Cnm-negative *S. mutans* strains MT8148, UA159, and GS5. Scale bar of the upper image, 500 nM; scale bar of the lower image, 200 nM; magnification of the upper image, ×80,000; magnification of the lower image, ×200,000.
