# Supplementary figures and images for: Cnm of Streptococcus mutans is important for cell surface structure and membrane permeability
Source: Front Cell Infect Microbiol. 2022 Sep 13;12:994014. doi: 10.3389/fcimb.2022.994014 (PMC9513430; doi:10.3389/fcimb.2022.994014)

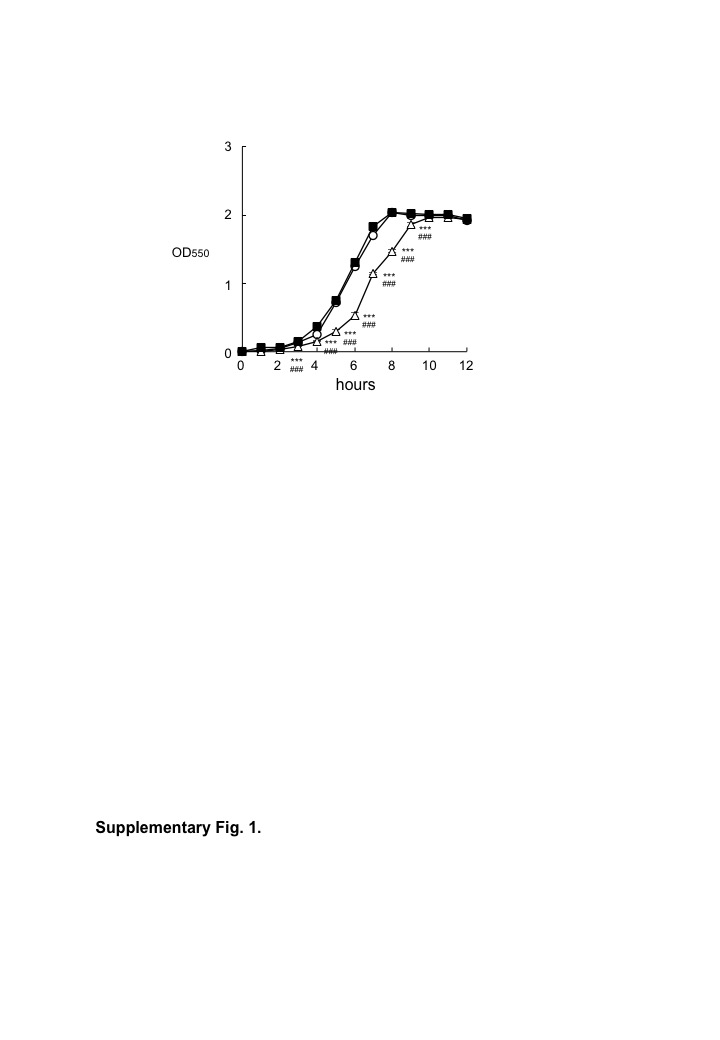

Supplement: Supplementary Figure 1 — Bacterial growth rates of strains SN74, SN74CND, and SN74CNDcomp. ○ SN74, Δ SN74CND, SN74CNDcomp. There were significant differences in the values between strains SN74 and SN74CND (***P < 0.001), and between strains SN74CNDcomp and SN74CND (### P < 0.001). Data are presented as the mean ± SD from five independent experiments. Statistical significance was determined using analysis of variance with Bonferroni’s correction. [file Image_1.jpeg]

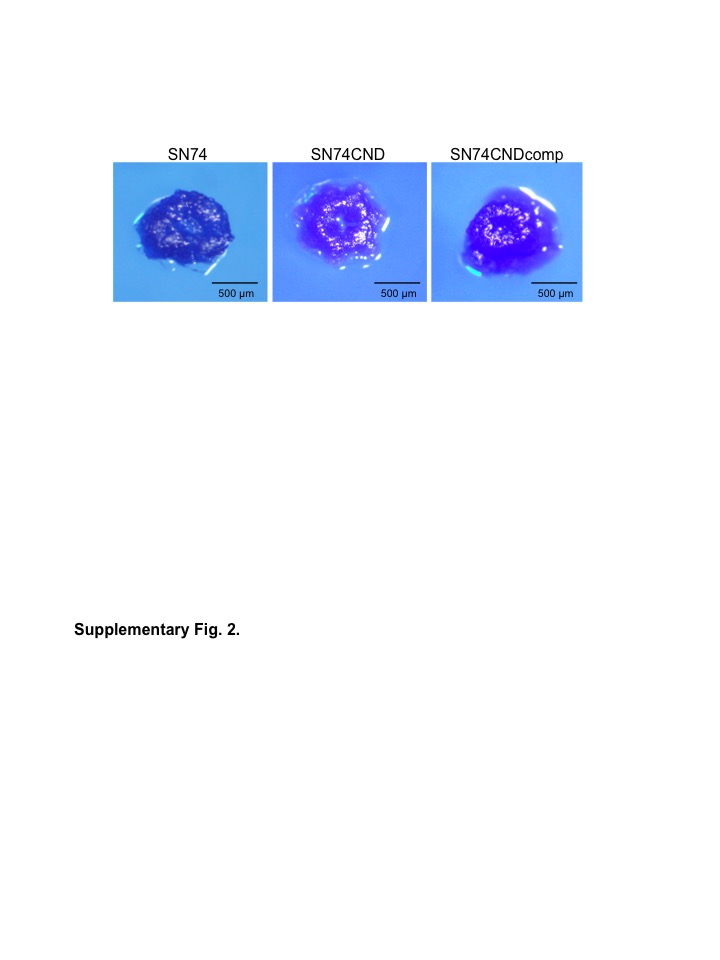

Supplement: Supplementary Figure 2 — Colony morphology of S. mutans strains SN74, SN74CND, and SN74CNDcomp. [file Image_2.jpeg]

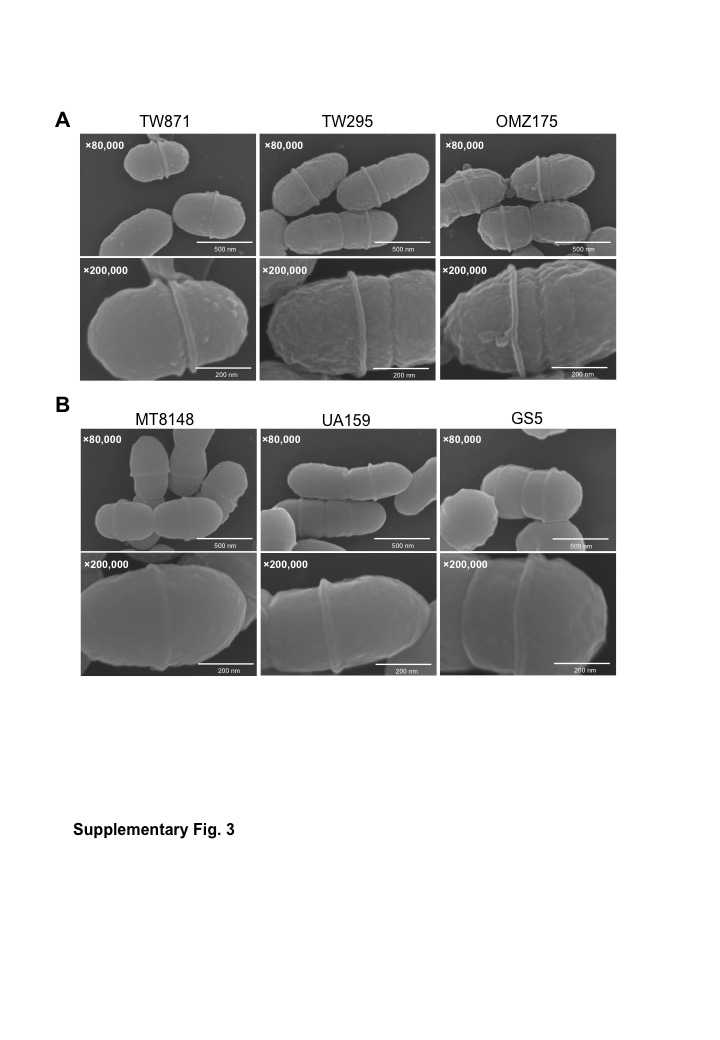

Supplement: Supplementary Figure 3 — Scanning electron microscopic images of S. mutans strains. A. Cnm-positive S. mutans strains TW871, TW295, and OMZ175. Scale bar of the upper image, 500 nM; scale bar of the lower image, 200 nM; magnification of the upper image, ×80,000; magnification of the lower image, ×200,000. B. Cnm-negative S. mutans strains MT8148, UA159, and GS5. Scale bar of the upper image, 500 nM; scale bar of the lower image, 200 nM; magnification of the upper image, ×80,000; magnification of the lower image, ×200,000. [file Image_3.jpeg]
